# From Moral Commitment to Systemic Neglect: Health System Perspectives on Preconception Care in Ethiopia From Healthcare Workers, Leaders, and Policymakers

**DOI:** 10.1111/phn.70164

**Published:** 2026-07-28

**Authors:** Yared Asmare Aynalem, Pauline Paul, Zohra S. Lassi, Salima Meherali

**Affiliations:** ^1^ Faculty of Nursing University of Alberta Edmonton Canada; ^2^ College of Health Science Debre Berhan University Debre Berhan Ethiopia; ^3^ Robinson Research Institute ,College of Health Adelaide University Adelaide Australia; ^4^ School of Public Health College of Health Adelaide University Adelaide Australia

**Keywords:** Ethiopia, health systems, interpretive description, policymakers, preconception care, qualitative research

## Abstract

**Objective(s):**

To explore how healthcare workers, managers, and policymakers understand, perceive, and operate preconception care (PCC).

**Design:**

An interpretive descriptive qualitative study was conducted (April–September 2025) in two government hospitals. Data from interviews, focus groups, document review, and observations were analyzed using constant‐comparative methods to generate an explanatory account of PCC practice.

**Sample:**

Thirty‐six participants were purposively recruited, including healthcare workers, hospital managers, policymakers, health bureau staff, and NGO stakeholders. Data were collected in Amharic, transcribed, translated, and analyzed inductively, focusing on patterns, tensions, and system‐level influences.

**Results:**

Preconception care emerged as structurally unsupported yet sustained through individual effort, shaped differently across system levels. Frontline providers delivered it through moral commitment and improvisation within routine services, rendering it invisible and inconsistent. Fragmented knowledge, shaped by a biomedical focus, limited training, and confusion with antenatal care, further undermined consistent delivery. Managers described the absence of training, supervision, and performance indicators, limiting accountability and integration into service delivery. Policymakers emphasized it as a strategic priority but highlighted gaps in operationalization, coordination, and resource allocation. Across levels, gendered norms and reliance on community trust networks shaped access and reinforced inequities, revealing a persistent misalignment between policy intent and practice.

**Conclusions:**

PCC functions as an informal practice sustained by individual agency within weakly institutionalized systems. Strengthening implementation requires bridging policy and practice through system integration, managerial accountability, and gender‐responsive, community‐engaged care models.

## 1 Introduction

Preconception care (PCC) is a cornerstone of reproductive, parental, and newborn health, emphasizing the improvement of parental well‐being before conception (Aynalem et al. 2025;2018; Dean et al. 2014; Gluckman et al., 2010; Stephenson et al. 2018; World Health Organization [WHO] 2013). It integrates biomedical, behavioral, and social interventions to identify and address risks before pregnancy, thereby enhancing parental outcomes, optimizing fetal development, and promoting long‐term population health (Gernand et al. 2016; Goossens et al. 2018; Lassi et al. 2014; Shawe et al. 2015; Stephenson et al. 2018). Parental health, shaped by nutrition, chronic disease control, emotional readiness, and environmental exposure, profoundly influences pregnancy outcomes and children's lifelong well‐being (Buklijas 2018; Dimitriadis et al. 2023; Fleming et al. 2021; Gernand et al. 2016; McAuliffe et al. 2020; Wadhwa et al. 2009). Strengthening PCC is thus both a parental health strategy and an intergenerational investment in disease prevention and health promotion (Gernand et al. 2016; Lassi et al. 2014; McAuliffe et al. 2020; Tekalign et al. 2021).

Despite growing recognition of its value, PCC remains inconsistently implemented across global health systems (Ayele et al. 2021; Frayne et al. 2016; Goossens et al. 2018; Lassi et al. 2014; Ukoha et al. 2022; WHO 2013). Many countries have incorporated PCC into their maternal and child health policies; however, its implementation in practice remains limited and uneven (Ayele et al. 2021; Dahab and Sakellariou, 2020); Shawe et al. 2015). Evidence across settings reveals common structural and professional barriers, limited access to services, low utilization, low awareness, limited education, socioeconomic disadvantage, and place of residence (Abayneh et al. 2022; Dimitriadis et al. 2023; Rono et al. 2022). Healthcare providers describe PCC as underdeveloped and poorly supported, acknowledged in policy but rarely prioritized in daily care (Dahab and Sakellariou 2020; Federal Democratic Republic of Ethiopia Ministry of Health [FMoH] 2024). Limited training, vague service mandates, weak interprofessional coordination, and the absence of monitoring frameworks continue to impede implementation (Federal Democratic Republic of Ethiopia Ministry of Health 2023a; Goossens et al. 2018; Shawe et al. 2015). Together, these challenges expose a persistent global divide between policy intent and operational reality (Ayalew et al. 2019a; Dahab and Sakellariou 2020). This divide is most visible in sub‐Saharan Africa, where health systems remain largely reactive and fragmented (Ayalew et al. 2019; Bauserman et al. 2020; Kamanga et al. 2022; Woldeyohannes et al. 2023). In Ethiopia, PCC has been integrated into the national reproductive, maternal, newborn, and child health (RMNCH) strategy, yet its uptake in public facilities remains limited (Abayneh et al. 2022; Federal Democratic Republic of Ethiopia Ministry of Health 2025). Existing studies, mainly quantitative, have focused on women's or healthcare providers’ knowledge, attitudes, and practices, consistently reporting low awareness and fragmented delivery (Ayalew et al. 2019; Federal Democratic Republic of Ethiopia Ministry of Health 2024). However, these descriptive accounts provide little insight into how institutional, professional, and policy factors shape the translation of PCC from guidelines to routine practice (Dahab and Sakellariou 2020; Woldeyohannes et al. 2023). Although national standards now exist, their effectiveness depends on how healthcare workers, leaders, and policymakers collectively interpret and implement them (Federal Democratic Republic of Ethiopia Ministry of Health 2023b). Providers enact policy through counseling, screening, and referral, while leaders and decision‐makers design the systems, training, supervision, and resource allocation that enable or constrain these actions (Ayele et al. 2021; Dahab and Sakellariou 2020; Federal Democratic Republic of Ethiopia Ministry of Health 2023a). Yet research has rarely examined these factors together, overlooking how their interdependent roles determine whether PCC becomes an integrated preventive service or remains peripheral to antenatal and delivery care (Ayalew et al. 2019).

This study addresses these gaps by examining how healthcare workers, leaders, and policymakers in Addis Ababa selected institutions conceptualize, perceive, and practice PCC within existing institutional and social contexts. It explores their knowledge, attitudes, and practices and analyzes how socioecological and intersecting factors, such as gender, culture, and institutional structures, shape the implementation of PCC in practice. Guided by an interpretive descriptive (ID) qualitative design informed by socioecological and intersectional perspectives, the study uncovers how PCC policies are enacted and identifies leverage points to strengthen system readiness, workforce engagement, and institutional coordination for equitable maternal health care in Ethiopia (Bronfenbrenner 1979; Crenshaw 2013; Thorne 2025).

## 2 Methods

### 2.1 Design and Methodological Orientation

In this study, we employed an Interpretive Description (ID) approach to explore how healthcare workers and institutional stakeholders understand, perceive, and operationalize PCC (Thorne 2025). ID is an applied qualitative methodology that generates clinically relevant, practice‐oriented insights through inductive and reflexive analysis grounded in context, enabling interpretation beyond description while remaining anchored in real‐world practice (Thorne 2025). A relativist ontology, interpretivist epistemology, and pragmatic orientation guided the study. The Socio‐Ecological Model and intersectionality theory served as sensitizing frameworks to interpret how multilevel and intersecting determinants influence PCC access, understanding, and delivery (Bronfenbrenner 1979; Crenshaw 2013).

### 2.2 Study Setting and Participant Recruitment

The study was conducted at two selected public hospitals that provide comprehensive maternal and reproductive health services. These institutions provided a rich context for exploring how PCC is understood and practiced at the clinical and leadership levels. Participants included frontline providers (nurses, midwives, physicians, public health officers), hospital leaders, PCC focal persons, program coordinators, and representatives from the Health Bureau and non‐governmental organizations. Eligibility requires at least 1 year of maternal or reproductive health experience and fluency in Amharic. Purposive sampling ensured diversity in role, gender, and seniority, while snowball sampling identified additional key informants at managerial and coordination levels. A face‐to‐face recruitment, facilitated through hospital leadership and the Health Bureau, continued until adequate *information power* was achieved (Malterud et al. 2016). Written informed consent was obtained in Amharic.

### 2.3 Data Collection

Data were collected between April 1 and September 14, 2025, using semi‐structured interviews, focus group discussions (FGDs), document review, and observation. Conducted in Amharic, interviews explored stakeholders’ understandings and experiences with PCC, while FGDs supported collective reflection. Guides were iteratively refined. Sessions lasted 30–90 min, were audio‐recorded, transcribed verbatim, and back‐translated into English. Field notes and analytic memos captured context and emerging insights. The Ethiopian PCC Guideline (Federal Democratic Republic of Ethiopia Ministry of Health 2024), service registers, and reporting templates were reviewed, and observations of maternal and child health units and workflows provided institutional context.

### 2.4 Data Analysis

Audio recordings were reviewed repeatedly to develop familiarity with participants’ accounts and to verify meanings against transcripts. Transcripts were coded in NVivo 15 through iterative engagement. Data collection and analysis occurred concurrently, consistent with the inductive logic of ID (Thorne 2025). Analysis began after the first two interviews and progressed alongside data generation using thematic analysis informed by grounded theory techniques adapted for applied inquiry(O'Callaghan et al. 2024; Thorne 2025). Line‐by‐line open coding identified concepts grounded in participants’ words and refined them through constant comparison within and across institutions. Axial coding organized related concepts into broader categories, and selective coding synthesized these into interpretive themes linking knowledge, attitudes, and practices with structural and socioecological influences. Triangulation strengthened analytic rigor by integrating interviews, focus groups, field observations, analytic memos, and document review (including the Ethiopian PCC Guideline and reporting templates). Interpretation followed Thorne's analytic strategies, pattern recognition, relational analysis, contextualization, and conceptual condensation guided by recursive questions: *What is happening? What does it mean? Why in this context? What practical insight does it offer?* This reflexive, iterative process ensured analytic depth, coherence, and applied relevance (Thorne 2025).

### 2.5 Rigor and Trustworthiness

Rigor followed interpretive description (ID) quality principles (Thorne 2025), ensuring epistemological integrity through alignment of aims, data sources, and interpretations. Credibility was enhanced by incorporating diverse clinical, administrative, and data perspectives across both hospitals. Analytic logic was maintained through a transparent audit trail of coding and memoing, while triangulation across interviews, focus groups, observations, and document review strengthened interpretive authority. The first author's insider–outsider position supported contextual insight while requiring ongoing reflexivity, sustained through journaling and supervisory dialogue. Thick description and illustrative quotations enhanced authenticity, resonance, and transferability. This research is also presented in accordance with the Standards for Reporting Qualitative Research (SRQR) checklist, as attached in Supporting Information 1.

### 2.6 Ethical Considerations

Ethical approval was obtained from the University of Alberta Research Ethics Board (Pro00149685_AME1) and the Addis Ababa Health Bureau (Ref. No: አ/አ/ጤ/2/198/17). Permission to collect data was granted by both participating hospitals. Written informed consent was obtained from all participants. Confidentiality was maintained through de‐identification, secure data storage, and restricted access to the research team. Participants were informed of their right to withdraw at any time without consequences. All interviews were conducted with cultural sensitivity and adherence to ethical research standards.

## 3 Results

### 3.1 Characteristics of Study Participants

A total of 36 participants took part in the study. Twenty‐four participants completed in‐depth individual interviews, including 18 healthcare workers and six leaders or decision‐makers, while 12 healthcare workers participated in focus group discussions. Participants were primarily recruited from two public healthcare institutions, and stakeholders represented governmental, non‐governmental, and partner organizations involved in maternal, reproductive, and preconception health. Healthcare workers included physicians (general practitioners, residents, and specialists), midwives, nurses, and public health officers. Stakeholders comprised hospital leaders, department heads, program directors, coordinators, advisors, and the PCC focal person. Participants’ ages ranged from 25 to 49 years, with the majority between 30 and 39 years, and professional experience ranged from 1 to over 16 years. Educational backgrounds varied and included bachelor's degrees (BSc), general medical qualifications (GP), master's‐level training (MPH, MSc, or RH), and specialist medical qualifications (see Table 1).

**TABLE 1:** Characteristics of study participants.

Characteristic	In‐depth interviews (*n* = 24)	Focus group discussions (*n* = 12)	Total (*n* = 36)
Participant category			
Healthcare workers	18	12	30
Stakeholders/Policy makers	6	—	6
Institution/Organization			
Institution 1	11	6	17
Institution 2	10	6	16
Addis Ababa Regional Health Bureau (AARHB)	1	—	1
John Snow Inc. (JSI)	1	—	1
Marie Stopes International Ethiopia	1	—	1
Sex			
Male	13	8	21
Female	11	4	15
Professional role/Position			
Physicians (GPs, residents, specialists)	8	6	14
Midwives	6	4	10
Nurses	4	2	6
Public health officer	1	—	1
Hospital leaders/Department heads	2	—	2
Program directors/Coordinators/Advisors	3	—	3
PCC focal person	1	—	1
Age group (years)			
25–29	4	2	6
30–34	6	4	10
35–39	6	4	10
40–44	4	1	5
45–49	4	1	5
Years of professional experience			
1–5 years	5	3	8
6–10 years	7	4	11
11–15 years	5	2	7
≥16 years	7	3	10
Highest educational qualification			
Bachelor's degree (BSc)	9	4	13
General practitioner (GP)	3	4	7
Resident physician	1	1	2
Specialist/Gynecologist (MD)	4	1	5
Master's degree (MPH/MSc/RH)	7	2	9

*Note*: All stakeholders and policy‐makers were recruited through in‐depth interviews. Focus group discussions were conducted only with healthcare workers. Age and experience are presented in grouped categories to preserve anonymity and align with qualitative reporting standards (COREQ/SRQR).

### 3.2 Overall Finding

This study identified six interrelated themes that explain how healthcare workers, leaders, and policymakers in selected hospitals in Addis Ababa understand and practice PCC within a system where it remains unestablished. Each theme is built on the insights and experiences of participants, forming a coherent narrative that moves from individual understanding to collective and systemic realities. The account begins with fragmented knowledge and preventive conviction, expands into shared and gendered responsibility and improvisation within fragile systems, and extends to community enablers and exclusions. It concludes with systemic neglect and emerging digital reforms, together illustrating how intertwined personal, cultural, and institutional influences shape the evolving practice and meaning of PCC. The main findings of the current study are also summarized below (See Figure 1).

**FIGURE 1:**
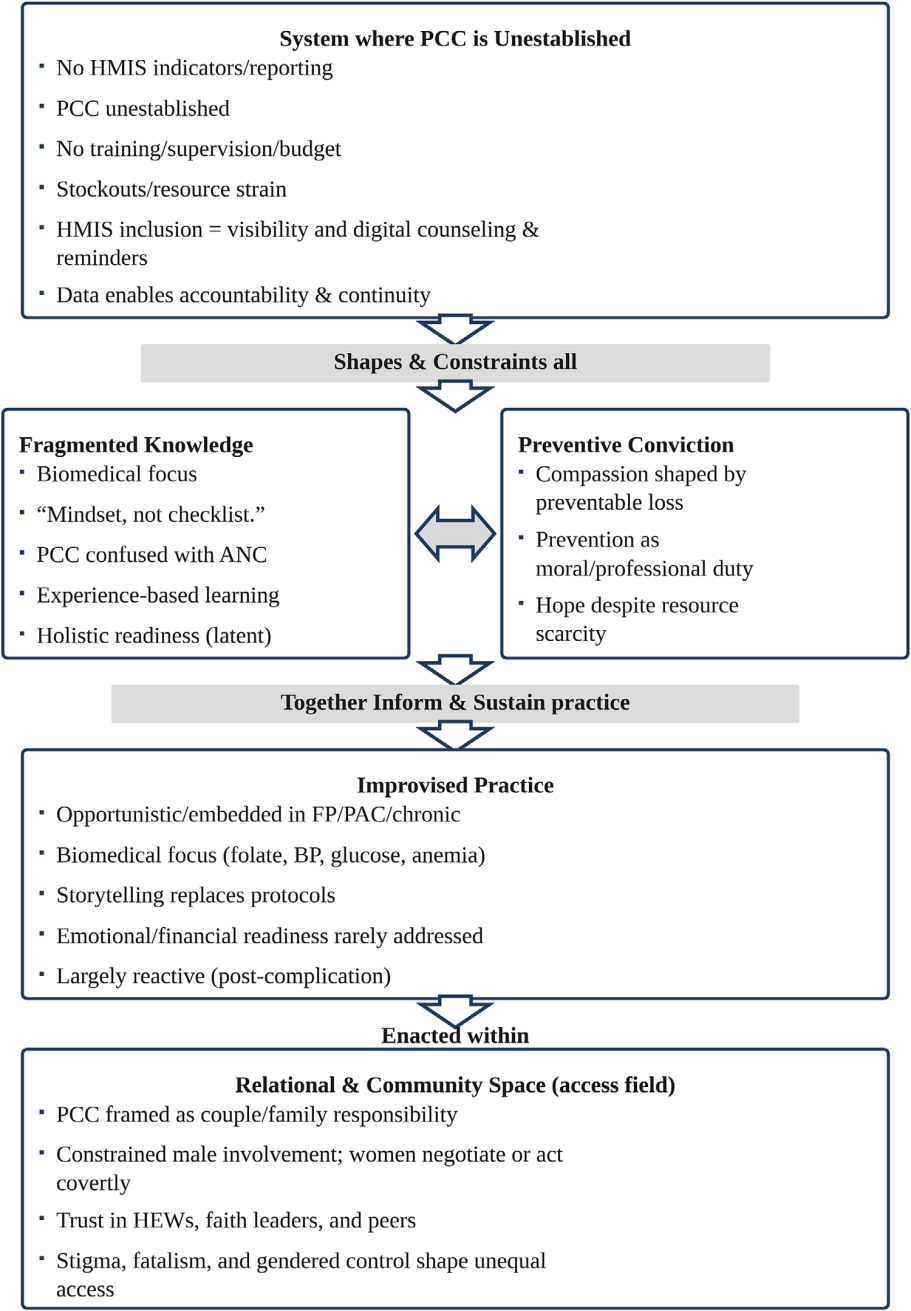
Interacting factors shaping preconception care practice among key health‐system stakeholders. [Colour figure can be viewed at wileyonlinelibrary.com]

#### 3.2.1 Fragmented Knowledge and System Gaps, With Digital/Data Infrastructures as Accelerators

Across participants, knowledge of PCC was described as broad yet fragmented, rooted in biomedical awareness but weakened by inconsistent training, conceptual confusion, and institutional neglect. While PCC was widely recognized as preventive, healthcare workers mainly framed it through screening and supplementation, whereas leaders linked its absence to system‐level invisibility. Many referred to PCC as “*preparation without a system*,” indicating an idea acknowledged but not implemented. Providers emphasized clinical tasks: “*We check blood pressure, sugar, and anemia before pregnancy… folic acid is important to prevent problems*” (HCW‐G06). Another added, “*It is the action taken before pregnancy happens… eight visits are recommended before pregnancy occurs*,” (HCW‐FGD1‐02), reflecting confusion with antenatal care. This fragmentation extended beyond clinical misunderstanding to the absence of structured implementation. As one healthcare worker described:

“In our country, most people have no idea about preconception care… I've heard of the concept, but we're not practicing it on the ground… we're not truly implementing it.” (HCW‐Z06)

While providers described gaps in practice, leaders interpreted these as consequences of system design, where services not embedded in reporting remain invisible.

A few participants recognized broader psychosocial and relational dimensions. “*We also have to consider fathers, because if the father drinks or smokes, it affects the baby*,” (HCW‐FGD2‐04). Others described readiness as emotional and financial: “*The woman should be ready mentally and physically… prepare financially and have reduced stress*,” (HCW‐G05). However, these views were not consistently applied, as most providers relied on experience rather than training. “*We did not get training on this; we just counsel based on our experience*,” (HCW‐Z05). Field observations confirmed no dedicated PCC rooms, guidelines, or supervision, indicating that broader understanding lacked structural support.Healthcare leaders emphasized invisibility as the key driver of neglect: “*If it is not part of the report, it is not part of the routine*,” (SH3). While providers highlighted daily constraints such as misinformation and stockouts, policymakers pointed to missing system components—training, supervision, digital integration, and budget. The 2024 guideline was described as comprehensive in theory but weak in implementation, leaving practice dependent on individual commitment.

Participants across groups identified digital and data integration as a way to institutionalize PCC. “*Once PCC enters the data system, it becomes real*,” said SH1. As one stakeholder explained:

“It should be fully integrated into HMIS… Without being part of HMIS, the data is fragmented and not prioritized… Once it enters the reporting system, it will live.” (SH6)

“*Without indicators, we cannot plan or improve*,” (SH4). Providers viewed digital tools more pragmatically: “*If the tool is digital, everyone gives the same message*” (HCW‐Z05). Technology was also seen as extending reach: “*We teach about folic acid and timing online; it reaches those who never come to the clinic*,” (HCW‐Z05).

Emerging efforts, such as appointing focal persons and piloting electronic checklists, suggested early steps toward integration. “*Even one dedicated corner gives PCC an identity*,” (SH2). Yet these remained limited. A policy advisor noted PCC was beginning to enter HMIS and CPD frameworks: “*Even one line in the system can change everything*,” (SH3). Overall, PCC knowledge exists but remains fragmented and differently understood across roles. Without guidelines, indicators, and HMIS integration, it remains a concept rather than routine care, with digital systems acting as a bridge to visibility, accountability, and sustainability.

#### 3.2.2 Preventive Attitudes Reframe PCC as Essential—Holistic Health

Across participants, prevention was framed as both moral conviction and professional identity, though expressed differently across roles. Healthcare workers emphasized prevention as part of everyday care and personal responsibility, while leaders framed it in terms of system efficiency and long‐term impact. It was described as “*a way of caring before crisis*,” grounded in compassion, responsibility, and dignity within a strained system. “*If we prepare before conception, we protect the next generation*,” explained HCW‐G04. Education was central: “*When women understand why this care matters, they follow through; it changes their whole attitude*” (HCW‐Z03). Prevention transformed reaction into readiness, linking clinical vigilance with relational well‐being. “*It is protection for the family, not just for the mother*,” reflected HCW‐G03.

Participants emphasized continuity of care across the reproductive journey. “*Preconception care clearly shows us how a woman… could get care before her conception… If one mother previously gave birth and had congenital abnormalities like spina bifida, we have to tell her where to get treatment… This also has an advantage for the country at large*” (HCW‐FGD1‐03). PCC was described as “*a mindset, not a checklist*,” connecting professional purpose with hope. Even amid scarcity, frontline providers expressed strong personal commitment: “*When mothers lose a baby because they did not get care early, it hurts me personally*” (HCW‐G07). “*Even without resources, I counsel because it saves lives*” (HCW‐G01). In contrast, leadership emphasized prevention as a system‐level strategy: “*When prevention is normalized, emergencies decrease, and costs are reduced*” (SH4). Yet observation revealed a gap between shared belief and system support. The 2024 national PCC guideline lacked operational follow‐up, training, budgeting, and concrete engagement strategies, leaving prevention largely dependent on individual motivation rather than institutional structure. Still, hope persisted across groups. “*If we teach consistently, the community will adapt*” (HCW‐G08). Prevention thus functioned as PCC's moral backbone, but also highlighted the limits of relying on values alone. As one provider concluded, “*Belief in prevention keeps us going, but belief alone is not enough; it must live in the system*” (HCW‐Z07).

#### 3.2.3 Relational and Gendered Shared Responsibility

Building on strong moral conviction, participants described PCC as a shared duty, though understood and enacted differently across groups. Healthcare workers emphasized shared responsibility within couples and everyday practice, while policymakers highlighted male involvement as a strategic gap in implementation. PCC was framed as a collective act linking couples, families, professionals, and communities. Preparation was seen as a joint responsibility. “*When men and women prepare together, both feel responsible; it is not only the woman's duty to be healthy*,” said HCW‐Z04. “*In the guideline, men are clearly mentioned… If men are not involved, women may not get the chance to use the service*” (SH6). HCW‐G09 affirmed, “*Excluding men means missing half the solution*.” This framing positioned shared accountability as essential but unevenly realized.

Responsibility also shaped professional identity. “*Even without training, we feel responsible to inform couples; if we do not, we have failed our duty*.” Yet this sense of duty was not institutionally sustained. Without indicators or budgets, collective responsibility remained informal. “*It should be fully integrated into HMIS… Without being part of HMIS, the data is fragmented and not prioritized*” (SH6). “*If it is not in the report, it's not routine*.” While the guideline acknowledged men, it lacked tools and accountability mechanisms. “*Most services are still focused on women… We need more strategies to engage men actively*” (SH6). Observations confirmed the absence of men‐friendly spaces and structured couple counseling, reinforcing that shared responsibility was more aspirational than operational.

Cultural analogies deepened the meaning of shared effort. Participants invoked debo (ደቦ) and ekub (እቁብ), community systems of mutual labor and financial support, as metaphors for PCC, emphasizing that preparation works best when responsibility is collective rather than individual. “*When we talk about health before pregnancy, it should be like community work, everyone contributes*,” (HCW‐Z08). However, entrenched gender norms constrained this ideal. “*If the husband insists, she becomes pregnant without PCC… many decisions about pregnancy are made by men*” (HCW‐FGD1‐06). Some women adapted quietly: “*I take folic acid quietly because if my husband finds out, it may cause problems*” (HCW‐FGD2‐04). Participants proposed outreach through radio, faith leaders, workplaces, and social media to normalize male involvement. “*When men hear it from church or their friends, it feels normal, not medical*.” Yet, as one leader emphasized, “*We cannot depend on personal effort forever; there must be system support*.” Relational responsibility thus bridged moral commitment and structural constraint: “*Preconception care is shared protection… When we all take responsibility, we all save lives*” (HCW‐Z01), but remained constrained by limited institutional support and persistent gender norms.

#### 3.2.4 Practices Shaped by Improvisation Under Fragile Systems

PCC existed not as a formalized program but as a fragile, improvised practice sustained by personal conviction rather than institutional design. Providers described it as “hidden but alive,” embedded informally within family‐planning, post‐abortion, and chronic‐care services. “*Actually, preconception care is not usual here in our context… we don't give preconception care as a separate service… our system is focused on managing confirmed diagnoses, not on prevention*” (HCW‐FGD1‐06). This improvisation reflected both professional creativity and systemic neglect: prevention endured without visibility, structure, or accountability. Field observations confirmed this fragmentation. Neither hospital had a designated PCC room, posted guidelines, or reporting tools. Counseling occurred in shared waiting areas or only after complications—folic acid advice during a family‐planning visit at one site, and only after miscarriage at another. The absence of space and protocol made PCC reactive rather than preventive, sustained quietly through individual effort.

Within this structural void, providers relied on empathy and relational trust. “*Many women come regularly for family planning… But we usually only talk about side effects and method choice. If we used that time to ask about their future pregnancy plans… Right now, that chance is missed*” (HCW‐FGD1‐05). Storytelling substituted for manuals, and counseling rarely extended beyond biomedical advice to emotional or financial readiness. Improvisation also shaped delivery models. “*This care is implemented in integrated ways… what is currently done is care is given to those who come to health institutions… But we are missing the larger community*” (SH6). Community actors and digital outreach partially filled institutional silence. Yet without formal reporting lines or system recognition, PCC remained invisible. “*Without integration into HMIS, PCC remains off the record and off the agenda*” (SH2). The national guideline, though conceptually comprehensive, lacked operational tools, supervision, costing, training, and data integration, rendering it rhetorical rather than actionable. Staff shortages and absent indicators left care dependent on conscience rather than policy, resilient, but structurally fragile.

#### 3.2.5 Community Enablers and Structural Exclusions—The Dual Face of Access

At the community level, PCC persisted through relationships rather than formal systems. Trust and moral framing were central. “*PCC only lives when people trust the messenger*.” Teachers, HEWs, elders, and faith leaders were described as the most credible voices. “*Culturally, our community respects advice from elders and religious leaders more than from health workers… if PCC is framed as a responsibility, not only as a medical service, people may accept it more*” (HCW‐FGD1‐04). Peer influence created a “chain of awareness.” “*When a woman hears it from someone she knows, she believes it*” (HCW‐G02). Providers stressed meeting people in churches, markets, schools, and workplaces. Health Bureau officers emphasized media: “*Local radio and TV are the strongest tools for rural and semi‐urban communities, while social media is key for youth in cities… Religious and community leaders should also be involved*” (SH6). Yet no PCC signage or materials were visible in facilities; counseling remained reactive, dependent on interpersonal trust. To reach excluded groups, participants proposed home visits, mobile clinics, weekend outreach, and digital platforms. Faith leaders framed preparation as protection. “*I don't think this is against community norms… PCC is counseling, treatment, and preparation*” (SH3). However, these same networks reinforced exclusion. Fatalism, “*If you plan pregnancy, the evil eye will follow you*”, and gender norms constrained autonomy. “*Many clients believe pregnancy is only God's decision… unless we frame it as God helping them protect the baby, they may reject the advice*” (HCW‐FGD1‐06). “*Even if she wants to come, she waits for her husband's permission*.” Unmarried and migrant women faced stigma; some used family planning secretly. Institutional silence deepened inequities. “*We report ANC and delivery, but PCC is nowhere… If it is not in the report, it doesn't exist*.” Without indicators, training, or supervision, prevention remained informal labor. Still, hope endured: “*Religion also teaches us to protect what God has given… families listen more*” (HCW‐FGD1‐04). Community ties functioned as both bridge and barrier, sustaining prevention while shaping who was seen and who remained excluded.

#### 3.2.6 Skepticism and Strain From Systemic Neglect

Healthcare workers’ strong moral commitment to prevention coexisted with eroding confidence in the system meant to sustain it. Participants described working “in the dark,” without manuals, reporting formats, or resources. “Actually, preconception care is not usual here… our system is focused on managing confirmed diagnoses, not on prevention” (HCW‐FGD1‐06). With no indicators or budget lines, PCC became “nobody's task,” surviving as invisible, voluntary labor rather than an institutional function. Field observations confirmed this fragility: neither hospital had a PCC unit, supervision structure, or consistent supplies. Providers improvised within family‐planning and post‐abortion services, often counseling only after complications occurred. As one participant stated, “You can't preach prevention when you have not eaten lunch” (HCW‐FGD2‐03). Prevention persisted “from the heart,” but the emotional toll was evident. “We see the same women coming again with complications; it hurts when you know it could have been prevented” (HCW‐FGD2‐05). Fatalistic beliefs, “Pregnancy is natural; why come before it happens?” deepened moral fatigue.

Resource scarcity intensified burnout. “We are told to promote PCC, but even folic acid is missing” (HCW‐FGD1‐01). Prevention remained unfunded and peripheral. “Without integration into HMIS, PCC remains off the record and off the agenda” (SH6). Bureau leaders acknowledged that only pilot programs had focal persons and supervision; most facilities lacked logistics, training, and referral pathways. Although a national PCC guideline existed, it lacked implementation mechanisms, costing, and accountability. Without integration into HMIS or continuing professional development (CPD), PCC remained structurally invisible. “It should be fully integrated into HMIS… Without being part of HMIS, the data is fragmented and not prioritized” (SH6). Yet skepticism reflected realism rather than resignation. “Once it enters the reporting system, it will live.” Another policy actor noted, “CPD and HMIS are the twin anchors; once PCC enters both, it becomes sustainable” (SH3). Providers sought validation more than reward: “We just need to be seen, supported, and trained, then prevention can finally start before the crisis.”

## 4 Discussion

This study examined how healthcare workers, leaders, and policymakers make sense of PCC within a context where prevention is morally valued but institutionally weak. The findings point to a central interpretive insight: PCC operates less as a formal program and more as a morally sustained practice, creating a persistent disjunction between what is believed, what is done, and what is structurally supported. While healthcare workers enact prevention through everyday care and personal responsibility, leaders and policymakers position its absence within failures of system visibility and accountability. This divergence is not simply a difference in perspective but reflects how location within the system shapes what counts as knowledge, responsibility, and action. Similar to findings from Ghana and Nigeria, biomedical framings remain dominant while psychosocial, gendered, and structural dimensions are less developed (Adjei et al. 2025; Gilson et al. 2017; Ojifinni & Ibisomi, 2020; Olajubu et al. 2025). However, rather than disengagement, participants in this study described compensatory practices, where moral commitment substitutes for institutional design. We interpret this condition as **resilient fragility**: a system that continues to function through ethical labor, yet remains structurally unstable (Woldeyohannes et al. 2023).

In the current study, knowledge of PCC is not simply incomplete but contextually produced. Healthcare workers rely on experiential, practice‐based knowing, while policymakers draw on abstract, guideline‐level knowledge that is weakly translated into action. This creates a disconnect between conceptual clarity and operational reality. Prevention is understood less as a set of standardized interventions and more as an ethical orientation, “care before crisis,” anchored in protecting future generations. In the absence of training, supervision, and accountability, knowledge becomes internalized rather than institutionalized. This supports the view that clinical knowledge in resource‐constrained settings evolves through contextual negotiation rather than protocol adherence (Thorne 2025). Although the 2024 national PCC guideline articulates a comprehensive vision, its limited implementation pathways render it symbolically present but practically absent (Federal Democratic Republic of Ethiopia Ministry of Health 2024). Thus, knowledge does not fail; rather, it shifts form, from system property to individual ethic.

This shift has consequences for how responsibility is organized. While PCC is framed as shared responsibility, our findings suggest that responsibility is redistributed rather than shared. Women disproportionately carry the burden of readiness, while men's involvement remains rhetorically endorsed but structurally unsupported. Healthcare workers attempt to bridge this gap through counseling and relational engagement, yet lack the institutional tools to do so consistently. In this way, gendered norms are not merely barriers but become embedded within how prevention is enacted. Consistent with prior studies (Hay et al. 2019; Kebede et al. 2023; World Health Organization 2024), male engagement remains underdeveloped; however, this study extends existing evidence by showing that moral appeals to shared responsibility, in the absence of structural mechanisms, can reinforce rather than disrupt inequity. Responsibility without power does not translate into agency.

Practice, in this context, is best understood as adaptive rather than programmatic. Healthcare workers integrate PCC into existing services, use informal communication strategies, and extend care beyond formal encounters. These practices demonstrate system responsiveness but also reveal how institutional gaps are absorbed into everyday work. Because PCC is not measured, supervised, or incentivized, this labor remains largely invisible. This aligns with broader evidence that health systems privilege what is counted, while overlooking relational and emotional work (Hill et al. 2020; World Health Organization 2021, 2024). The implication is not only under‐recognition, but also the normalization of moral labor as a substitute for system responsibility. Over time, these risks sustain services at the cost of equity and provider well‐being (Stephenson et al. 2018). At the community level, PCC is similarly shaped by relational dynamics. Trust enables engagement where formal systems are weak, positioning community actors as critical extensions of care. However, this reliance on social networks introduces conditional access. Participation is mediated by norms of respectability, marital status, and social belonging, which can exclude already marginalized groups. This reinforces the understanding of PCC as a relational process, where access is negotiated through social legitimacy as much as clinical need (Aynalem et al. 2025; Gilson et al. 2017). Thus, the same mechanisms that enable reach can simultaneously reproduce inequity.

Digital integration emerges as a potential mechanism to shift PCC from moral practice to institutionalized care, but only if interpreted beyond its technical function. For policymakers, HMIS integration represents visibility, accountability, and legitimacy; for healthcare workers, digital tools offer standardization and practical support. This dual meaning highlights that digital systems are not neutral, they reorganize what is seen, valued, and acted upon. Consistent with existing literature (Erku et al. 2023; Labrique et al. 2018), measurement can transform hidden practices into recognized system functions. However, without attention to access and equity, digitalization risks reproducing existing exclusions (Ndemo 2025). Taken together, these findings suggest that the core challenge in PCC implementation is not awareness but misalignment between moral commitment and institutional structure. Prevention is sustained through people, relationships, and values, while systems remain oriented toward reactive care. This creates a form of structural dissonance in which belief exceeds capacity. An interpretive reading of these findings suggests that strengthening PCC requires more than introducing guidelines or tools; it requires reconfiguring how prevention is valued, measured, and supported within the system. Embedding PCC into supervision, financing, training, and reporting is not simply a technical adjustment but a shift in what counts as legitimate care. Only when moral labor is recognized and institutionalized can prevention move from fragile endurance to sustainable and equitable practice.

### 4.1 Implications of the Study

Sustaining PCC requires linking moral commitment with structural reform. The 2024 national PCC guideline should move from design to implementation by integrating PCC into HMIS, supervision, and financing systems to ensure visibility and accountability. Policies must promote gender‐ and couple‐responsive approaches, and health extension workers, teachers, and faith leaders should be trained and supervised as trusted community advocates. In practice, providers need training, supervision, a separate room, and CPD to embed PCC within family planning and chronic care services, supported by digital tools for counseling and reporting. Future research should test integrated PCC models that connect the guidelines' priorities with digital systems, CPD, and community outreach. Aligning conviction with structure will make PCC a sustainable public health service.

### 4.2 Strengths and Limitations

A key strength of this study is its multilevel qualitative design, incorporating perspectives from policymakers, leaders, and healthcare providers to provide a comprehensive understanding of institutional and professional influences on PCC. Triangulation and the use of ID, informed by socioecological and intersectional perspectives, strengthened analytic rigor and contextual depth. Limitations include potential loss of nuance during translation from Amharic to English, limited transferability beyond two urban sites, and the evolving national PCC reforms that may affect the long‐term applicability of findings.

## 5 Conclusion

In the study area, PCC is sustained more by individual moral commitment than by system design. Although healthcare workers, leaders, and policymakers express strong preventive values, PCC remains fragmented and informal due to limited institutional support, weak accountability, and poor integration into routine services. As a result, prevention relies on personal initiative rather than structured implementation. Achieving equitable and sustainable PCC requires shifting from individual effort to institutional ownership by embedding PCC within data systems, supervision, training, financing, and gender‐ and community‐responsive structures. Aligning ethical commitment with structural support is essential for PCC to become a core health‐system function.

## Author Contributions

*Conceptualization*: Yared Asmare Aynalem, Pauline Paul, and Salima Meherali. *Methodology*: Yared Asmare Aynalem, Pauline Paul, and Salima Meherali. *Formal analysis*: Yared Asmare Aynalem. *Investigation*: Yared Asmare Aynalem. *Writing – original draft*: Yared Asmare Aynalem. *Writing – review & editing*: Pauline Paul, Salima Meherali, and Zohra S. Lassi. *Supervision*: Pauline Paul, Salima Meherali, and Zohra S. Lassi. *Funding Acquisition*: Yared Asmare Aynalem. *Software*: Yared Asmare Aynalem.

## Funding

This research was supported by the Vanier Canada Graduate Scholarship and the International Development Research Centre (IDRC) through the IDRC Doctoral Research Award. The funders had no role in the study design, data collection, analysis, interpretation, or manuscript preparation.

## Conflicts of Interest

The authors declare no conflicts of interest.

## Ethics Statement

Ethical approval was obtained from the University of Alberta Research Ethics Board (Pro00149685_AME1) and the Addis Ababa Health Bureau (Ref. No: አ/አ/ጤ/2/198/17). Permission to collect data was granted by both participating hospitals. Written informed consent was obtained from all participants. Confidentiality was maintained through de‐identification, secure data storage, and restricted access to the research team. Participants were informed of their right to withdraw at any time without consequences. All interviews were conducted with cultural sensitivity and adherence to ethical research standards.

## Supporting information




**Supporting File 1**: phn70164‐sup‐0001‐SuppMat.docx

## Data Availability

Data supporting the findings of this study are available from the corresponding author upon reasonable request. Data are not publicly available due to ethical and confidentiality constraints.
